# Medical and genetic correlates of long-term buprenorphine treatment in the electronic health records

**DOI:** 10.1038/s41398-023-02713-x

**Published:** 2024-01-10

**Authors:** Maria Niarchou, Sandra Sanchez-Roige, India A. Reddy, Thomas J. Reese, David Marcovitz, Lea K. Davis

**Affiliations:** 1 Vanderbilt Genetics Institute, Vanderbilt University Medical Center, Nashville, TN USA; 2 Department of Psychiatry, University of California San Diego, San Diego, CA USA; 3 Division of Genetic Medicine, Department of Medicine, Vanderbilt University Medical Center, Nashville, TN USA; 4 Department of Psychiatry and Behavioral Sciences, Vanderbilt University Medical Center, Nashville, TN USA; 5 Department of Biomedical Informatics, Vanderbilt University Medical Center, Nashville, TN USA; 6 Department of Molecular Physiology and Biophysics, Vanderbilt University, Nashville, TN USA

**Keywords:** Addiction, Predictive markers

## Abstract

Despite the benefits associated with longer buprenorphine treatment duration (i.e., >180 days) (BTD) for opioid use disorder (OUD), retention remains poor. Research on the impact of co-occurring psychiatric issues on BTD has yielded mixed results. It is also unknown whether the genetic risk in the form of polygenic scores (PGS) for OUD and other comorbid conditions, including problematic alcohol use (PAU) are associated with BTD. We tested the association between somatic and psychiatric comorbidities and long BTD and determined whether PGS for OUD-related conditions was associated with BTD. The study included 6686 individuals with a buprenorphine prescription that lasted for less than 6 months (short-BTD) and 1282 individuals with a buprenorphine prescription that lasted for at least 6 months (long-BTD). Recorded diagnosis of substance addiction and disorders (Odds Ratio (95% CI) = 22.14 (21.88–22.41), *P* = 2.8 × 10^−116^), tobacco use disorder (OR (95% CI) = 23.4 (23.13–23.68), *P* = 4.5 × 10^−111^), and bipolar disorder (OR(95% CI) = 9.70 (9.48–9.92), *P* = 1.3 × 10^−91^), among others, were associated with longer BTD. The PGS of OUD and several OUD co-morbid conditions were associated with any buprenorphine prescription. A higher PGS for OUD (OR per SD increase in PGS (95%CI) = 1.43(1.16–1.77), *P* = 0.0009), loneliness (OR(95% CI) = 1.39(1.13–1.72), *P* = 0.002), problematic alcohol use (OR(95%CI) = 1.47(1.19–1.83), *P* = 0.0004), and externalizing disorders (OR(95%CI) = 1.52(1.23 to 1.89), *P* = 0.0001) was significantly associated with long-BTD. Associations between BTD and the PGS of depression, chronic pain, nicotine dependence, cannabis use disorder, and bipolar disorder did not survive correction for multiple testing. Longer BTD is associated with diagnoses of psychiatric and somatic conditions in the EHR, as is the genetic score for OUD, loneliness, problematic alcohol use, and externalizing disorders.

## Introduction

Buprenorphine, which is a partial opioid agonist, is one of three medications approved by the U.S. Food and Drug Administration (FDA) for the treatment of opioid use disorder (OUD) [1]. Studies indicate that buprenorphine treatment is associated with reduced opioid use [2] and higher treatment retention rates [3] compared with placebo. Moreover, patients undergoing buprenorphine treatment have a lower risk for all-cause and overdose mortality [4] and fewer opioid-related emergency department visits [5]. A retrospective longitudinal study, of adult Medicaid patients with OUD who had filled buprenorphine prescriptions for a minimum of 6 months before discontinuing refills, found that patients with longer buprenorphine duration treatment (more than 15–18 months) were less likely to be hospitalized, be seen in an emergency department, or receive a prescription for an opioid analgesic [6]. Moreover, there is no upper threshold on how long a person may benefit from buprenorphine, and patients may be counseled to stay on buprenorphine indefinitely. Despite the benefits of buprenorphine, retention in buprenorphine treatment remains poor [7], and between 50% to 80% of patients discontinue treatment within 6 months [8].

Previous research on the impact of co-occurring somatic, psychiatric, and other substance use disorders on buprenorphine treatment duration (BTD) has yielded mixed results. For example, studies across different settings found that higher retention was either not associated with comorbidity profiles [9], associated with lower rates of psychiatric diagnoses [10, 11], or associated with greater odds of retention [12]. The differences in these findings may be related to study design and setting. For example, in retrospective studies that rely on EHR data, diagnosis of mental health conditions may indicate help-seeking behavior on the part of the patient, and treatment for the diagnosis on the part of the provider, both of which may influence BTD independent of the presence of a mental health condition. Similarly, these associations may be explained by the heightened utilization of healthcare services among patients undergoing buprenorphine treatment which facilitates the diagnosis and treatment of other conditions.

Another factor that may differentiate patients who are retained in buprenorphine treatment is the genetic liability for physical and mental health conditions. For example, if patients with a higher genetic liability for OUD are more likely to be retained in buprenorphine versus another medication, this can help guide the doctors’ decision-making in terms of which medication treatment may be more appropriate for the patient. There are now large genome-wide association studies (GWAS) for OUD [13], as well as conditions closely related to OUD, including chronic pain [14], problematic alcohol use (PAU) [15], depression [16], loneliness [17], externalizing psychopathology [18], cannabis use disorder [19], nicotine dependence [20] and bipolar disorder [21]. Taking into account that thousands of genetic variants of small effect sizes contribute to complex conditions, we examined whether the cumulative effect of common alleles associated with the conditions described above (also known as polygenic scores, or PGS) contributes to the variation in BTD.

To increase buprenorphine retention rates and treatment effectiveness for OUD, further research is needed to identify and understand factors that are associated with buprenorphine treatment duration. Thus, the overall aim of our study was to better understand the characteristics of patients who stay in buprenorphine treatment for at least 6 months compared to those who stop treatment earlier than 6 months (BTD) [22]. To address our aim, we examined 1) the associations between somatic and psychiatric comorbidities and BTD using data from VUMC’s electronic health record (EHR), and 2) whether PGS for OUD, chronic pain, PAU, depression, loneliness, externalizing psychopathology, nicotine dependence, cannabis use disorder, and bipolar disorder were associated with BTD.

## Subjects and methods

### Electronic health record data

The Vanderbilt University Medical Center (VUMC) EHR was established in 1998 [23] and houses the medical records of 3.2 million individuals. The Synthetic Derivative (SD) database is a de-identified mirror image of the VUMC EHR and includes data on billing codes from the International Classification of Diseases, 9th and 10th editions (ICD-9 and ICD-10), demographics, Current Procedural Terminology (CPT) codes, laboratory values, clinical documentation, and prescription medications. In 2007 VUMC launched a biobank (BioVU) that stores DNA from over 250,000 VUMC patients, and links genotype data to the SD [24]. The BioVU sample included 66,914 patients (56% females, current age at the time of the analysis (SD) = 59.95 (22.5), of European Ancestries). This study was reviewed and approved by the VUMC institutional review board (IRB) for exemption from informed consent requirements (IRB approval: 201203).

### Buprenorphine treatment duration

We identified individuals with at least one prescription of buprenorphine between the years 1997 to 2019 and categorized them into (a) patients with buprenorphine prescription duration of less than 6 months (short-BTD) and (b) patients with buprenorphine prescription duration of equal to or greater than 6 months (long-BTD). This categorization was based on evidence suggesting that patients retained in treatment beyond 6 months show improved outcomes [5, 6]. Because we lacked information regarding the end date of the buprenorphine prescription, we employed a pragmatic approach by considering the standard 30-day duration for pharmacy fills. To classify patients into the long-BTD group, we established a criterion of requiring at least six buprenorphine prescriptions within a 6-month interval that was calculated as the time between consecutive prescriptions. The buprenorphine-related prescriptions are listed in Supplementary Table 1. To enrich the population of patients for whom VUMC was the most likely source of primary care, we used a heuristic ‘medical home’ definition (i.e., at least five codes on different days over a span of 3 years) [25]. We chose this approach to reduce confounding due to systematic missing values in the data. We also removed from the sample all individuals who had at least one cancer-related ICD-code, taking into account that cancer-related pain may be related to long-term analgesic use. Finally, although the vast majority of buprenorphine products used in VUMC are for OUD, there are two buprenorphine products that are used for pain in the outpatient clinics by the pain medicine physicians (i.e., Belbuca and Butrans). Fourteen percent (14%) of the total sample had received this prescription, and we also removed these individuals from our analyses. This resulted in a total of 6686 patients with short-BTD and 1282 patients with long-BTD.

### Genotyping

We restricted our genetic analyses to patients of European ancestry. Principal components of genetic variation were estimated to define patients of European ancestries. We did not run our analyses in patients with African Ancestry, due to a low total sample size (*n* = 83). Five hundred and forty-seven genotyped individuals of European ancestry received buprenorphine prescriptions and were used in the PGS analyses. Genotyping was performed using the Illumina MEGA_ex platform. The Michigan Imputation Server and the Haplotype Reference Consortium reference panel were used for the genotype imputation. Single nucleotide polymorphisms (SNPs) with good imputation quality (*R*^2^ > 0.3) were converted to hard calls. PGS analysis was restricted to autosomal chromosomes. We applied an Identity By Descent filter of 0.2 to exclude related individuals. For more details on the genotyping and quality control see [26].

### Polygenic scores (PGS)

We generated PGS for every patient in BioVU by training the PGS on the latest publicly available GWAS for OUD (*N*_effective_(*N*_eff_) = 74,646) [13], chronic pain [14] (*N*_eff_ = 387,649), PAU [15] (*N*_eff_ = 300,789), loneliness [17] (*N*_eff_ = 445,024), depression [16] (*N*_eff_ = 500,199), externalizing psychopathology [18] (*N*_eff_ = 1,492,085), nicotine dependence [20] (*N*_eff_ = 243,783), cannabis use disorder [19] (*N*_eff_ = 306,240), and bipolar disorder [21] (*N*_eff_ = 392,324) at the time of the analysis. The selection of PGS traits was determined by considering traits that exhibited a strong genetic correlation with OUD in previous studies, as well as traits for which there were well-powered GWAS available. PGS was calculated using the PRS-CS software [27] with the ‘auto’ training parameter, which applies a continuous shrinkage (CS) prior to SNP effect sizes. PGS was standardized for each analysis to have a mean of 0 and a standard deviation of 1.

### Statistical analyses

#### Phenome-wide association study (PheWAS)

We performed a PheWAS in 7968 individuals to identify the psychiatric and somatic diagnoses that may be associated with BTD. The phenotypes included were based on mapping International Classification of Disease versions 9 and 10 (ICD-9 and ICD-10) codes to 1,817 phecodes as described and validated by the Phecode Map 1.2b1 [28]. Specifically, phecodes are hierarchical categorizations of ICD-9 and ICD-10 codes, after extensive consultations from experts in a variety of medical fields. The phecodes have a standardized vocabulary and can refer to diseases, traits or symptoms (http://phewascatalog.org). We required a minimum of 100 cases for each phecode. A cases was defined if they had at least 2 ICD codes on different days. We fitted 334 logistic regressions (the remaining 1483 phenotypes had <100 cases) to test the association between dichotomized “long” and “short” BTD (predictor variable) and each phecode (outcome) after adjusting for the median age of EHR record, race, ethnicity, sex, insurance type and year of treatment. To account for multiple testing in the PheWAS analysis we used a Bonferroni corrected threshold of (*p* < 0.05/334 = 1.4 × 10^−4^). BTD was not treated as a continuous measure due to the large positive skewness of the variable. As a sensitivity analysis, we further adjusted for other pain-related phenotypes. We also performed a PheWAS to identify the medical conditions between the presence and absence of a buprenorphine prescription. Specifically, we fitted 1723 logistic regressions to test the association between ‘presence’ and ‘absence’ of buprenorphine prescription (predictor variable) and each phecode (outcome) after adjusting for median age of EHR record, race, ethnicity and sex. To account for multiple testing in the PheWAS analysis we used a Bonferroni corrected threshold of (*p* < 0.05/1723 = 2.9 × 10^−5^).

#### PGS analyses

For PGS analyses, we conducted 18 logistic regressions in which the outcome variable was (1) absence vs presence of at least one buprenorphine prescription or (2) short vs long BTD. Each of the PGS (i.e., OUD, chronic pain, PAU, loneliness, depression, externalizing disorders, nicotine dependence, cannabis use disorder, bipolar disorder) served as the predictor variables, controlling for sex, current age, and 10 principal components of ancestry. The models were the following:

1 $${\rm{Buprenorphine}}({\rm{yes}}/{\rm{no}}) \sim {\rm{PGS}}+{\rm{age}}+{\rm{sex}}+10{\rm{PCs}}$$

2 $${\rm{Buprenorphine}}({\rm{short}}/{\rm{long}}) \sim {\rm{PGS}}+{\rm{age}}+{\rm{sex}}+10{\rm{PCs}}$$

We used a Bonferroni corrected threshold of (*p* < 0.05/18 = 0.0028). Considering that OUD, loneliness, and chronic pain are genetically correlated, we further adjusted the analyses of the PGS of OUD and BTD for the PGS of chronic pain, and the analysis of the PGS of loneliness and BTD, for the PGS of OUD.

## Results

### Sample characteristics

Our sample included 7968 individuals with at least one buprenorphine prescription: 6686 had a treatment duration that lasted <6 months (short-BTD)(57% females, mean median age of 41.3 (16.0) at the record), and 1282 had a buprenorphine prescription that lasted at least 6 months or longer (long-BTD) (66% females, mean median age at record = 41.9 (11.9)). Thirty-four percent of the total sample had at least one OUD ICD code, including 84% of individuals in the long-BTD group and 24% of individuals in the short-BTD group (Table 1). This demonstrates that OUD was not consistently coded in the EHR while it was present and being treated.

**Table 1:** Demographics of the sample included in the Phenome Wide Association Study.

Demographics	Buprenorphine treatment duration	
	Less than 6 months (short-BTD)	At least 6 months (long-BTD)
*N*	6686	1282
Median age of record (Mean (SD)	41.3 (16.0)	41.9 (11.9)
Sex	3843 females (57%)	844 females (66%)
Race	6032 white, 458 black, 77 Asian	1225 white, 45 black, 2 Asian
Year of treatment (median)	2008	2013
*Insurance type*		
Private	4704 (70%)	587 (46%)
Public	1840 (28%)	674 (53%)
Third-party	36 (0%)	8 (0%)
Unknown	106 (0%)	13 (0%)
Presence of pain-related ICD code	4437 (66%)	841 (66%)
Presence of OUD-related ICD code	1637 (24%)	1075 (84%)
Number of buprenorphine prescriptions (mean(sd))	2.4 (3.6)	36.9 (37.6)
Participants with only one buprenorphine prescription(*N*/%)	4352 (65%)	NA

### PheWAS of BTD

**Fig. 1:**
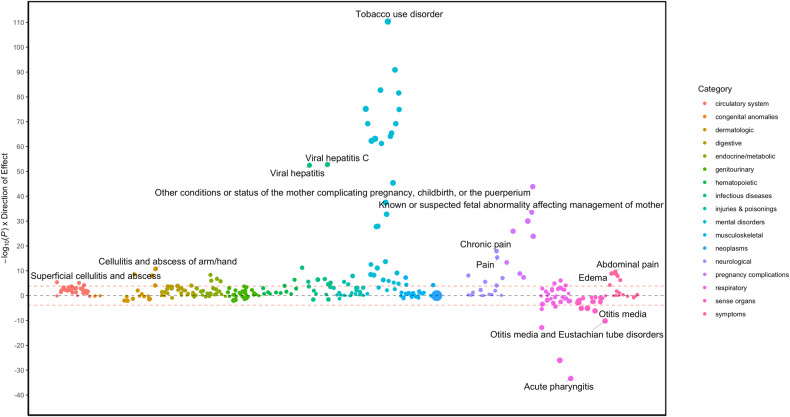
Manhattan plot of pheWAS results of Buprenorphine Treatment Duration (<6 months vs. equal or more than 6 months). The *y*-axis shows the −log10 transformed *p*-values multiplied by the direction of effect. The dots represent the phecodes which are grouped along the *x*-axis by phecode category. The colors of the dots indicate the phecode categories, which are explained in the figure. The dotted red line signifies the Bonferroni-corrected threshold for statistical significance.

Eighty medical diagnoses were associated with BTD (*p* < 1.4 × 10^−4^), of which 9 were negatively associated with BTD (i.e., presence of a diagnosis was associated with short BTD) (Table 2, Supplementary Table 2, Fig. 1). Substance addiction and disorders (OR(95% CI) = 22.14(21.88–22.41), *P* = 2.8 × 10^−116^), tobacco use disorder (OR(95% CI) = 23.40 (23.13–23.68), *P* = 4.5 × 10^−111^), bipolar disorder (OR(95% CI) = 9.70 (9.48–9.92), *P* = 1.3 × 10^−91^), mood disorders (OR(95% CI) = 6.68 (6.48–6.87), *P* = 2.64 × 10^−82^), and post-traumatic stress disorder (OR(95% CI) = 10.01 (9.77–10.24, *P* = 1.9 × 10^−83^) diagnoses were associated with longer BTD. On the other hand, acute illness such as acute sinusitis (OR(95% CI) = 0.10 (0.00–0.52, *P* = 8.8 × 10^−27^), acute pharyngitis (OR(95% CI) = 0.16 (0.00–0.46, *P* = 4.4 × 10^−34^), allergic rhinitis (OR(95% CI) = 0.23 (0.00–0.62, *P* = 1.4 × 10^−13^), cough (OR(95% CI) = 0.58 (0.36–0.81, *P* = 3.8 × 10^−6^), different types of otitis media including otitis media and eustachian tube disorders (OR(95% CI) = 0.16 (0.00–0.71, *P* = 6.50 × 10^−11^) suppurative and unspecified otitis media (OR(95% CI) = 0.17 (0.00–0.94, *P* = 7.5 × 10^−6^) and acute upper respiratory infections of multiple or unspecific sites (OR(95% CI) = 0.17 (0.00–0.40, *P* = 2.2 × 10^−49^) were associated with shorter BTD.

**Table 2:** Primary PheWAS analyses: strongest BTD associations per phecode group according to effect size.

Phenotype	Group	OR	Lower 95% CI	Upper 95% CI	*p*
Tobacco use disorder	Mental disorders	23.40	23.13	23.68	4.5 × 10^−111^
Chronic airway obstruction	Respiratory	1.98	1.67	2.28	1.3 × 10^−5^
Viral hepatitis C	Infectious diseases	4.27	4.08	4.45	1.7 × 10^−53^
Infectious and parasitic complications affecting pregnancy	Pregnancy complications	10.41	10.02	10.81	9.9 × 10^−31^
Edema	Symptoms	2.26	2.00	2.53	1.2 × 10^−9^
Osteomyelitis, periostitis, and other infections involving bone	Musculoskeletal	2.72	2.36	3.08	5.6 × 10^−8^
Chronic pain	Neurological	2.33	2.14	2.52	1.3 × 10^−18^
Suppurative and unspecific otitis media	Sense organs	0.17	0.00	0.94	7.5 × 10^−6^
Poisoning by antibiotics	Injuries & Poisonings	2.81	2.46	3.17	1.3 × 10^−8^
Cellulitis and abscess of arm/hand	Dermatologic	2.72	2.43	3.01	1.7 × 10^−11^
Essential hypertension	Circulatory system	1.50	1.33	1.68	4.1 × 10^−6^
Hypopotassemia	Endocrine/metabolic	1.85	1.59	2.10	1.7 × 10^−6^
Other anemias	Hematopoietic	1.68	1.48	1.89	4.3 × 10^−7^

We focused on analyzing the respiratory phenotypes and their relationship with the timing of diagnosis in relation to the first buprenorphine prescription. Among the total sample, 65% had at least one respiratory-related ICD code. Among these individuals, 71% of the respiratory diagnoses were observed in the short-term BTD group, while only 35% occurred in the long-term BTD group. For the long-term BTD, we found that 47% of the respiratory-related diagnoses occurred after the first buprenorphine prescription, 7% were diagnosed on the same day, while 46% of the diagnoses were made prior to their first buprenorphine prescription. Conversely, for the short-term BTD group, the pattern was different. Here, 51% of the respiratory diagnoses were observed after the first buprenorphine prescription, with 42% occurring on the same day. Notably, only for 7% of the individuals in the short-term BTD group, the respiratory diagnosis was made before their first buprenorphine prescription.

**Fig. 2:**
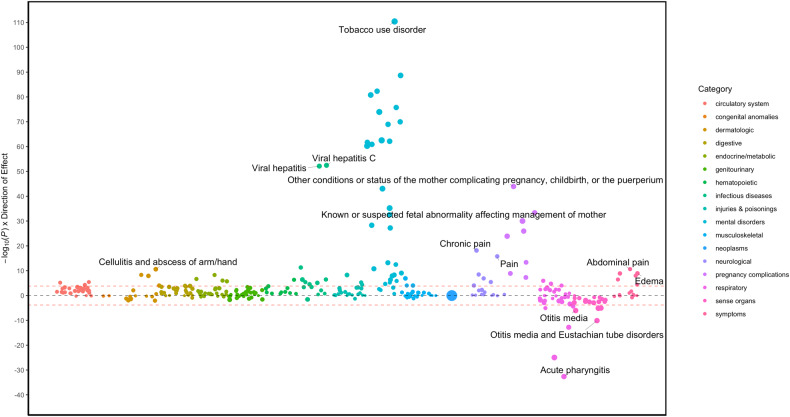
Manhattan plot of pheWAS results of Buprenorphine Treatment Duration (<6 months vs. equal or more than 6 months) adjusting for pain-related phenotypes. The *y*-axis shows the −log_10_ transformed p-values multiplied by the direction of effect. The dots represent the phecodes which are grouped along the *x*-axis by phecode category. The colors of the dots indicate the phecode categories, which are explained in the figure. The dotted red line signifies the Bonferroni-corrected threshold for statistical significance.

As expected, pain-related disorders were also positively associated with BTD, including chronic pain (OR(95% CI) = 2.33 (2.14–2.52, *P* = 1.25 × 10^−18^), pain (OR(95% CI) = 1.99 (1.82–2.15, *P* = 4.2 × 10^−16^), and acute pain (OR(95% CI) = 1.84(1.64–2.05, *P* = 8.2 × 10^−09^). After conditioning the PheWAS for chronic pain, all prior associations remained significant, and virtually unchanged (Fig. 2, Supplementary Table 3).

### PheWAS of absence/presence of buprenorphine prescription

Our sample included 7968 individuals with at least one buprenorphine prescription and 980,041 controls. There were 427 associations that surpassed the Bonferroni significance threshold (*p* < 2.9 × 10^−5^) (Supplementary Table 4). Among the strongest associations in terms of effect size were mental disorders during/after pregnancy (OR(95% CI) = 39.85(39.71–39.98), *p* < 1.13 × 10^−304^), substance addiction and disorders (OR(95% CI) = 33.74 (33.69–33.98), *p* < 1.13 × 10^−304^), suicidal ideation (OR(95% CI) = 17.50(17.41– 17.58), *p* < 1.13 × 10^−304^), posttraumatic stress disorder (OR(95% CI) = 17.29(17.21–17.37), *p* < 1.13 × 10^−304^), and viral hepatitis C (OR(95% CI) = 16.37(16.29–16.46), *p* < 1.13 × 10^−304^).

### PGS analyses

#### Absence vs. presence of at least one buprenorphine prescription

There were 547 patients with genetic data and at least one buprenorphine prescription (65% females, mean age(SD) = 50.9(15.1)) and 66,367 controls (i.e., patients with genetic data, without a buprenorphine prescription) (56% females, current mean age (SD) = 60.0(22.5)). The PGS of OUD as well as most OUD comorbid conditions that we examined were associated with the presence of a buprenorphine prescription in a patient’s EHR (Table 3). Specifically, the PGS of OUD was significantly associated with the presence of buprenorphine (OR per SD increase in PGS = 1.18 (1.09–1.29), *P* = 0.0001), as was the PGS of chronic pain (OR = 1.15 (1.06–1.25), *P* = 0.001), problematic alcohol use (OR = 1.17 (1.08–1.28), *P* = 0.0002), externalizing disorders (OR = 1.32 (1.21–1.44), *P* = 1.5 × 10^−10^), cannabis use disorder (OR = 1.17 (1.07–1.28), *P* = 0.0005), and bipolar disorder (OR = 1.14 (1.04–1.24), *P* = 0.004). On the other hand, the correlation between any buprenorphine prescription and PGS of loneliness did not survive correction for multiple testing (*P* < =0.0028) (OR = 1.08 (0.99–1.17), *P* = 0.09), and neither did the PGS of nicotine dependence (OR = 1.03 (0.95–1.13, *P* = 0.41), bipolar disorder (OR = 1.14 (1.04–1.24, *P* = 0.004) and depression (OR = 1.13 (1.04–1.23), *P* = 0.004). The variance explained ranged between 0.09% and 0.7% (Table 3).

**Table 3:** Results of the associations between BTD and PGS.

Buprenorphine presence (*N* = 547)/absence (*N* = 66,367)			
Trait	OR (95% CI)	*P*	Nagelkerke’s *R*^2^ (%)
Opioid use disorder	**1.18 (1.09–1.29)**	**0.0001**	**0.25**
Chronic pain	**1.15 (1.06–1.25)**	**0.001**	**0.2**
Problematic alcohol use	**1.17 (1.08–1.28)**	**0.0002**	**0.2**
Loneliness	1.08 (0.99–1.17)	0.09	0.05
Depression	1.13 (1.04–1.23)	0.004	0.1
Externalizing	**1.32 (1.21–1.44)**	**1.5** **×** **10**^**−10**^	**0.7**
Nicotine dependence	1.03(0.95–1.13)	0.41	0.01
Cannabis use disorder	**1.17(1.07–1.28)**	**0.0005**	**0**
Bipolar disorder	1.14(1.04–1.24)	0.004	0.1
**Buprenorphine <6 months (*N*** = **414) vs. at least 6 months (*N*** = **133)**			
**Trait**	**OR (95% CI)**	***P***	**Nagelkerke’s** ***R***^**2**^ **(%)**
Opioid use disorder	**1.43(1.16–1.77)**	**0.0009**	**3.2**
Chronic pain	1.35 (1.10–1.67)	0.0049	2.2
Problematic alcohol use	**1.47 (1.19–1.83)**	**0.0004**	**3.6**
Loneliness	**1.39 (1.13–1.72)**	**0.002**	**2.8**
Depression	1.21 (0.98–1.48)	0.07	0.9
Externalizing disorders	**1.52 (1.23–1.89)**	**0.0001**	**4.2**
Nicotine dependence	0.93 (0.76–1.13)	0.46	0.2
Cannabis use disorder	1.32(1.06–1.64)	0.01	1.8
Bipolar disorder	1.31(1.06–1.62)	0.01	1.8

In bold are associations that have surpassed the Bonferroni correction *p*-value (*p* < =0.05/18 = 0.0028).

#### Short vs. long BTD

Out of the 547 patients with genetic data and at least one buprenorphine prescription, 414 patients had a buprenorphine prescription that lasted less than 6 months (66% females, mean current age = 52.1(15.8), and 133 patients with a buprenorphine prescription that lasted more or equal to 6 months (59% females, mean current age = 47.2(12.1)).

The PGS for OUD (OR per SD increase in PGS(95% CI) = 1.43 (1.16–1.77, *P* = 0.0009), problematic alcohol use (OR(95% CI) = 1.47 (1.19–1.83, *P* = 0.0004), loneliness (OR(95% CI) = 1.39 (1.13–1.72, *P* = 0.002) and externalizing disorders (OR(95% CI) = 1.52 (1.23–1.89, *P* = 0.0001) were significantly associated with long-BTD (Table 3). There was weaker evidence that the PGS of chronic pain (OR(95% CI) = 1.35 (1.10–1.67, *P* = 0.005), cannabis use disorder (OR = 1.32 (1.06–1.64), *P* = 0.01), and bipolar disorder (OR = 1.31 (1.06–1.64), *P* = 0.01) were associated with long-BTD (Table 3). We tested whether the significant effects of PGS on BTD were accounted for by the PGS of OUD. We found that the association remained significant for the PGS of loneliness (OR_loneliness_ = 1.39 (1.13–1.71), *P* = 0.002), the PGS of PAU (OR_PAU_ = 1.43 (1.15–1.78), P = 0.001), and the PGS of externalizing disorder (OR_ext_ = 1.50 (1.21 to 1.86), *P* = 0.0003). There was no evidence for associations with the PGS of depression, nicotine dependence, and long-BTD (Table 3).

## Discussion

In a sample of over 7,000 individuals with buprenorphine prescription data extracted from EHR, we tested the associations between somatic and psychiatric comorbidities and BTD. Presence of a buprenorphine prescription was associated with over 420 medical diagnoses, including, as expected, substance use-related disorders. There were also 80 coded medical diagnoses associated with long-BTD (a buprenorphine prescription for at least 6 months) compared to short-BTD (a buprenorphine prescription for less than 6 months). When we further covaried for pain-related codes, the associations remained virtually unchanged. We also found that higher PGS for OUD, chronic pain, PAU, externalizing, and cannabis use disorder were related to the presence of a buprenorphine prescription, while the PGS for OUD, problematic alcohol use, loneliness, and externalizing disorders were associated with long BTD.

Coded diagnoses of mood disorders, post-traumatic stress disorder, suicidal ideation, pain-related conditions and substance use disorders were among the strongest associations with long-BTD. These findings support the notion that patients receiving longer BTD treatment may also be more likely to receive medical care for comorbid mental health conditions. The presence of a coded diagnosis in the EHR is indicative of clinical attention to a specific mental or physical health need and a course of clinical treatment or management. Thus, clinical diagnosis and supportive management of mental health conditions may be associated with longer BTD treatment. Of note, these findings also replicate previous studies showing that comorbid substance use [10, 29, 30], depression [30], and other psychiatric disorders [12], including suicidal behavior or thinking [31, 32] are related to retention in treatment, while absence of pain [32] is related to lower likelihood of retention. Furthermore, there is some indication that buprenorphine may have anti-depressant properties [33–35]. Lastly, as previously suggested [32], patients seeking treatment for psychiatric disorders may also be more experienced with mental health treatment. This experience could potentially enhance their understanding of mental health management and increase their likelihood of longer buprenorphine treatment therapies. This aligns with the notion that patients with more severe phenotypes and co-occurring disorders may require longer buprenorphine treatment to effectively address their complex healthcare needs.

Notably, respiratory-related diagnoses were negatively associated with long-BTD. Our analysis yielded two key points related to this finding. Firstly, a significantly higher percentage of individuals in the short-term BTD group had respiratory-related diagnoses compared to the long-term BTD group (71% vs. 35%). Secondly, the timing of respiratory diagnoses differed between the two groups. In the short-term BTD group, a considerable portion of individuals (42%) received their respiratory diagnosis on the same day as their first buprenorphine prescription, while in the long-term BTD group, only 7% received such a diagnosis on the same day. A possible explanation for this result is that those in the short-term BTD group were lower healthcare utilizers such that they did not receive a diagnosis prior to presenting to care for buprenorphine initiation. Furthermore, the proportion of individuals receiving a respiratory diagnosis after their first prescription was similar in both groups (51% vs. 47%), which would be unexpected if respiratory issues were caused by buprenorphine or leading to discontinuation of treatment.

There were also some seemingly unexpected associations with long-term BTD, such as pregnancy-related complications and poisoning by antibiotics. The presence of a specialized clinic at Vanderbilt, focusing on the prenatal and postnatal care of women with opioid use disorder, could account for the association between long-term BTD and pregnancy-related complications. Moreover, pregnant females have higher adherence and duration of treatment compared to males and other females, and although buprenorphine can improve outcomes, this population is still at higher risk for infectious diseases from IV drug use. Severe bacterial infections can occur at the site of injection and some antibiotics that are used can cause toxicity if not monitored correctly. Therefore, this association is not likely directly linked to long-term BTD but rather reflects correlations with bacterial infections, which may be due to IV drug use.

OUD is frequently comorbid with alcohol use [36], depression [37], and chronic pain [36], and is also considered an externalizing disorder. Moreover, OUD is a heritable disorder that is also found to be genetically correlated to major depressive disorder [13], bipolar disorder [38], cannabis and nicotine dependence [39], and alcohol dependence [13]. Higher PGSs for OUD, chronic pain, PAU, externalizing disorders, and cannabis use disorders were associated with the presence of a buprenorphine prescription. However, the associations between the PGS of nicotine dependence, depression, and bipolar and the presence of a buprenorphine prescription did not surpass the Bonferroni correction threshold. This lack of associations may be due to the relatively lower statistical power of GWAS conducted on nicotine dependence and bipolar disorder. The genetic scores for OUD, PAU, loneliness, and externalizing disorders were also correlated with a longer duration of buprenorphine treatment. These associations remained significant even after adjusting for the PGS of OUD, indicating that these associations are less likely to be explained by the common genetic variation between OUD and these traits. Conversely, the associations between the genetic scores for depression, cannabis use disorder and bipolar disorder and long-BTD did not surpass the Bonferroni correction threshold, possibly due to low power.

Notably, while the PGS for loneliness was not associated with the presence or absence of buprenorphine prescription, it was associated with long-BTD. This finding indicates that genes involved in perceptions of social connectedness may not influence the decision to start buprenorphine, but may sensitize people to the benefits of longer treatment duration. It is also possible that this finding was a Type I error. Future studies are needed to replicate this finding and also to test whether the genes tagged in the loneliness PGS modify the response to buprenorphine.

### Study limitations

One limitation of the study is that the VUMC’s Biobank is not representative of the general population. Because enrollment in the biobank is secondary to a routine clinical blood draw, biobank participants tend to have higher healthcare usage, to be older, and to be insured. Although we examined a sample that is enriched for patients who receive primary care in VUMC, missing data and limited prescription information are also limitations in our study. Furthermore, as VUMC is an open system, patients may receive care outside of VUMC. Another limitation of our study is the absence of data regarding the dosage and end dates of buprenorphine prescriptions. This lack of information could have compromised the precision of our phenotypic classification. However, it is important to emphasize that this limitation primarily affects the statistical power of our analysis. Moreover, the distributional properties of BTD in our sample did not allow us to examine BTD as a continuous variable, but this may be feasible in different samples where the distribution of BTD may exhibit different characteristics. Also, the sample size of our African Ancestry population as well as other non-EU ancestry populations was too small to conduct genetic analyses. It is important for future studies to examine whether our results can be replicated in the African American ancestry sample. Finally, although the PheWAS is an established methodology, more fine-tuned hypothesis-driven specific phenotyping could be a future direction (e.g., clinical report studies to delve into particular associations suggested by PheWAS).

## Conclusions

In a large sample of patients with a buprenorphine prescription, we identified 80 coded medical diagnoses that were associated with a longer duration of buprenorphine treatment. We found that higher PGS for OUD and OUD-related conditions were associated with the presence of a buprenorphine prescription. Finally, among patients with a buprenorphine prescription, higher PGS for OUD, PAU, loneliness and externalizing disorders were related to a longer buprenorphine treatment duration. Future studies are needed to replicate our findings in other cohorts and also further explore the nature of these associations and potential mechanisms implicated in BTD.

### Supplementary information

Supplementary material

## Data Availability

Due to data sharing restrictions related to privacy concerns in the Electronic Health Records, the datasets generated from our hospital population will not be publicly available.
